# Prevention of cervical cancer through two HPV-based screen-and-treat implementation models in Malawi: protocol for a cluster randomized feasibility trial

**DOI:** 10.1186/s40814-021-00839-7

**Published:** 2021-04-20

**Authors:** Jennifer H. Tang, Jennifer S. Smith, Shannon McGue, Luis Gadama, Victor Mwapasa, Effie Chipeta, Jobiba Chinkhumba, Erik Schouten, Bagrey Ngwira, Ruanne Barnabas, Mitch Matoga, Maganizo Chagomerana, Lameck Chinula

**Affiliations:** 1 Department of OB-GYN, University of North Carolina, 4002 Old Clinic Building, CB #7570, Chapel Hill, NC 27599-7570 USA; 2 University of North Carolina Project-Malawi, Lilongwe, Malawi; 3 Lineberger Comprehensive Cancer Center, University of North Carolina, Chapel Hill, NC USA; 4 Department of Epidemiology, University of North Carolina, Chapel Hill, NC USA; 5 College of Medicine, University of Malawi, Blantyre, Malawi; 6 Management Sciences for Health, Lilongwe, Malawi; 7 Centre for Health, Agriculture, Development Research, and Consulting, Blantyre, Malawi; 8 Department of Global Health, University of Washington, Seattle, WA USA

**Keywords:** Cervical cancer, Screening, Self-sampling, HPV testing, Family planning, Community, Thermal ablation, Sub-Saharan Africa, Malawi, Implementation

## Abstract

**Background:**

Cervical cancer is the leading cause of cancer incidence and mortality among Malawian women, despite being a largely preventable disease. Implementing a cervical cancer screening and preventive treatment (CCSPT) program that utilizes rapid human papillomavirus (HPV) testing on self-collected cervicovaginal samples for screening and thermal ablation for treatment may achieve greater coverage than current programs that use visual inspection with acetic acid (VIA) for screening and cryotherapy for treatment. Furthermore, self-sampling creates the opportunity for community-based screening to increase uptake in populations with low screening rates. Malawi’s public health system utilizes regularly scheduled outreach and village-based clinics to provide routine health services like family planning. Cancer screening is not yet included in these community services. Incorporating self-sampled HPV testing into national policy could address cervical cancer screening barriers in Malawi, though at present the effectiveness, acceptability, appropriateness, feasibility, and cost-effectiveness still need to be demonstrated.

**Methods:**

We designed a cluster randomized feasibility trial to determine the effectiveness, acceptability, appropriateness, feasibility, and budget impact of two models for integrating a HPV-based CCSPT program into family planning (FP) services in Malawi: model 1 involves only clinic-based self-sampled HPV testing, whereas model 2 includes both clinic-based and community-based self-sampled HPV testing. Our algorithm involves self-collection of samples for HPV GeneXpert® testing, visual inspection with acetic acid for HPV-positive women to determine ablative treatment eligibility, and same-day thermal ablation for treatment-eligible women. Interventions will be implemented at 14 selected facilities. Our primary outcome will be the uptake of cervical cancer screening and family planning services during the 18 months of implementation, which will be measured through an Endline Household Survey. We will also conduct mixed methods assessments to understand the acceptability, appropriateness, and feasibility of the interventions, and a cost analysis to assess budget impact.

**Discussion:**

Our trial will provide in-depth information on the implementation of clinic-only and clinic-and-community models for integrating self-sampled HPV testing CCSPT with FP services in Malawi. Findings will provide valuable insight for policymakers and implementers in Malawi and other resource-limited settings with high cervical cancer burden.

**Trial registration:**

ClinicalTrials.gov identifier: NCT04286243. Registered on February 26, 2020.

## Background

Cervical cancer is largely preventable through screening and preventive treatment (CCSPT), yet it remains the leading cause of cancer cases and cancer deaths among Malawian women [1]. Resource-limited countries bear a disproportionate share of global cervical cancer burden: incidence and mortality are two to four times higher in resource-limited countries compared with the highest-resource countries [2]. However, innovative methods for screening and treatment offer increased opportunities to scale-up CCSPT in resource-limited countries.

Malawi introduced a national CCSPT program in 2004 to work towards a national screening rate of 80%, but its reach has been limited. As of 2015, only 26.5% of women had been screened at least once in their lifetime, and only 43% who screened positive received appropriate treatment [3]. The currently used primary screening strategy (visual inspection with acetic acid or VIA) requires a time-consuming pelvic examination, which is challenging to offer for large scale screening because of limited clinic space at health facilities and limited numbers of providers. Timely preventive treatment of screen-positive women is also difficult to achieve because cryotherapy, the currently used ablative treatment, requires relatively expensive and heavy refrigerant gas cylinders [3]. Many of these barriers apply in other resource-limited settings beyond Malawi.

Since the implementation of Malawi’s CCSPT program, the World Health Organization (WHO) now recommends human papillomavirus (HPV)-based screen-and-treat programs where feasible [4]. HPV assays have improved sensitivity over VIA, although with somewhat lower specificity for the detection of high-grade cervical dysplasia [5, 6]. Newer HPV assays allow for results within hours and perform similarly whether a self-collected or provider-collected cervicovaginal sample is used. Self-sampling can remove key bottlenecks for screening because it eliminates the need for a provider and an examination table for initial screening and may be more acceptable to women than physician sampling [7]. A meta-analysis of 34 studies showed that offering self-sampling increased screening uptake compared to standard-of-care, though most of the studies were from high-resource countries [8]. HPV viral DNA remains stable on dry cervicovaginal swabs up to a month after collection [9, 10], which allows delayed testing of self-collected samples and opens up the opportunity for community-based screening, which is especially important for women in rural areas.

Meanwhile, thermal ablation provides a less resource-intensive method for treating screen-positive women. Similar to cryotherapy, thermal ablation is simple to learn and can be performed by mid-level providers, but it does not require heavy gas cylinders and is available through hand-held portable devices. Early experience suggests that thermal ablation has high cure rates, similar to cryotherapy and loop electrosurgical excisional procedures (LEEP) [11, 12]. Some facilities in Malawi have already pivoted to using thermal ablation due to the difficulties with maintaining functioning cryotherapy machines [3, 13].

Introducing a screen-and-treat strategy that combines these innovations—self-sampling, rapid HPV assays, and thermal ablation—promises to improve screening and treatment rates in resource-limited settings including Malawi. However, success requires considering key implementation questions around how to target healthy women and how to reach rural women who have poor access to health facilities. Integrating cervical cancer screening with family planning (FP) services is a promising strategy because many women who access FP services are in the appropriate screening age range and sexually-active. Existing structures to deliver community-based FP services can be utilized to reach women in rural and isolated areas. Any solution for increasing CCSPT services in sub-Saharan Africa must be applicable to rural populations, since an estimated 60–75% of women who develop cervical cancer in sub-Saharan Africa live in rural areas [14].

In many countries, community health workers provide a vital link between health facilities and rural populations. Community-based FP services have been effective in increasing FP uptake in many countries [15], including in Malawi where 80% of the population is rural [16, 17]. Community health workers in Malawi, known as health surveillance assistants (HSAs), provide FP services to rural women through outreach and village clinics. Experience from other countries suggests that community health workers like HSAs can be successfully trained to offer self-sampling for HPV screening in rural areas [18, 19]. However, providing community-based screening introduces additional challenges for follow-up given that same-day screening and treatment will not be conducted. Understanding the impact of community-based screening on loss to follow-up is important given that one of the primary shortcomings in Malawi’s CCSPT program has been the low rate of treatment among screen-positive women. Thermal ablation may be more scalable than cryoablation but would only improve access if women are successfully informed of their results and linked to care at facilities.

We designed a cluster randomized trial to evaluate the effectiveness, acceptability, appropriateness, feasibility, and cost impact of two implementation models. In model 1 (clinic-only), self-sampling will be offered to eligible women attending visits at health facilities (especially targeting those attending FP visits), with same-day thermal ablation for HPV-positive, ablation-eligible women. In model 2 (clinic + community), the same screen-and-treat algorithm will be offered at health facilities, and community-based screening will be offered through HSAs, with facility referral of HPV-positive women.

## Methods/design

### Aims and objectives

Our aim is to compare the effectiveness, acceptability, appropriateness, feasibility, and budget impact of integrating self-sampled HPV testing into FP services via clinics only versus including community-based screening.

Our specific objectives are as follows:

1. Compare the effectiveness of our two models on uptake of CCS and FP services among eligible women in the catchment areas of health facilities assigned to the two models. The effectiveness will be evaluated through analysis of an Endline Household Survey, which will be administered to randomly selected eligible women in the catchment areas of the health facilities at the end of project implementation.
  - We hypothesize that the proportion of eligible women who have received CCS services during the 12 months of project implementation will be higher among eligible women in the catchment areas of health facilities assigned to model 2 than in model 1.
  - We hypothesize that the proportion of eligible women who have received FP services during the 12 months of project implementation will be higher among eligible women in the catchment areas of health facilities assigned to model 2 than in model 1.
2. Assess the acceptability, appropriateness, and feasibility of the two models among key stakeholders, including providers, facility management teams, and clients. These implementation outcomes will be evaluated through analysis of mixed methods assessments, including use of quantitative tools (Acceptability-Appropriateness-Feasibility Tool, Client Exit Survey) as well as qualitative interviews with providers, facility management teams, and clients.
  - We hypothesize that both models will be viewed as acceptable and appropriate by health care providers, facility management teams, and clients.
  - We hypothesize that both models will be feasible in a variety of health facilities in Malawi.
3. Estimate the cost and budget impact of each model compared to the standard-of-care (VIA and cryotherapy). These outcomes will be evaluated through analysis of structured observations during time-and-motion studies, the Workload Assessment Tool, and cost expenditures during implementation of the study.
  - We hypothesize that both models will be more cost-effective than the current standard-of-care for the number of cervical cancer case averted.
  - We hypothesize that model 1 will be more cost-effective than model 2.

### Design

**Fig. 1:**
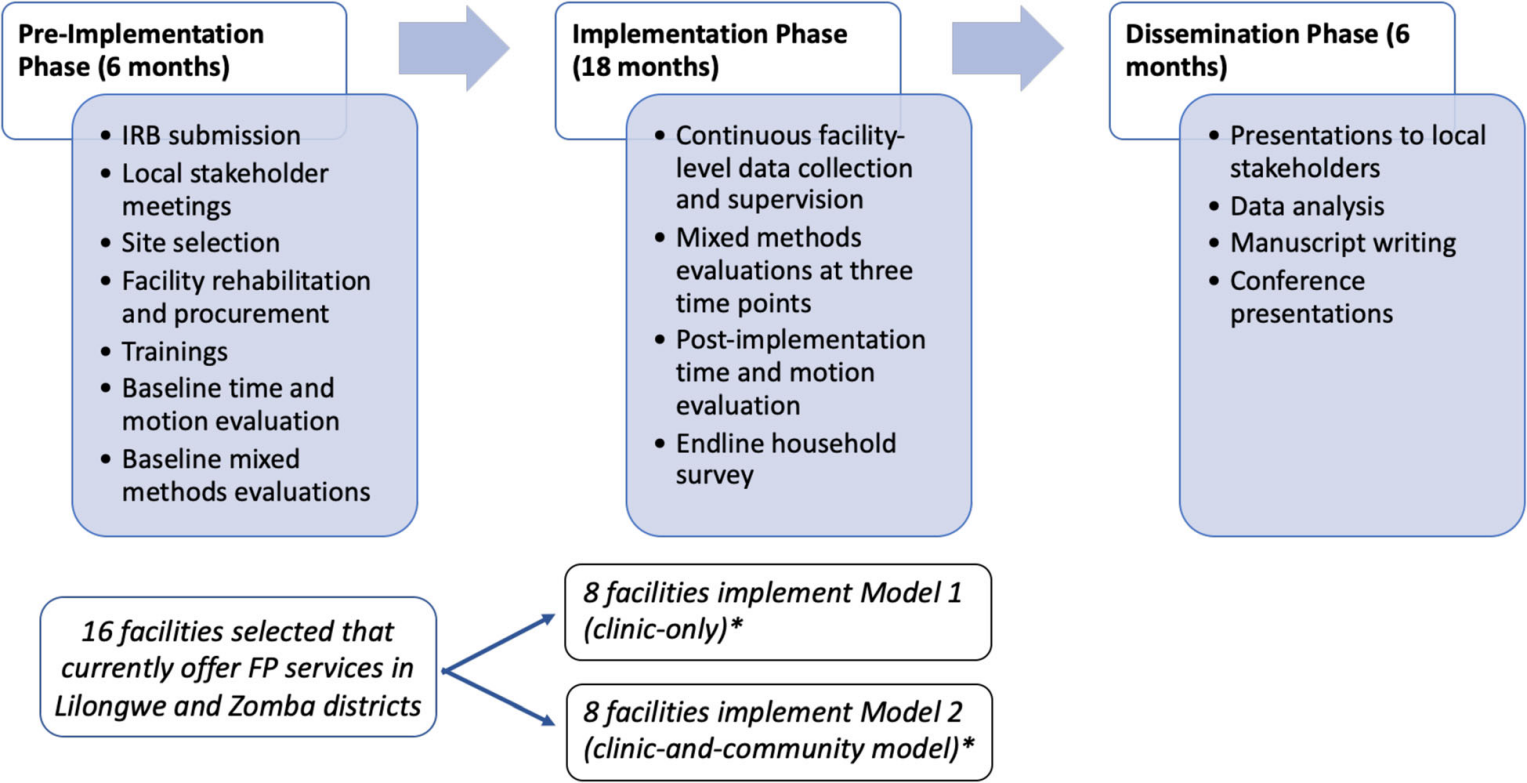
Study phases, with activities listed for each phase. Italicized portion indicates site selection and implementation of the two models under the appropriate phases. *Due to constraints on community services offered at two matched facilities, these facilities were both assigned to model 1 and dropped from randomization scheme. Thus, 9 facilities will implement model 1 and 7 will implement model 2

This is a hybrid type 2 cluster randomized feasibility trial, with 16 health facilities in Malawi assigned to either a clinic-only or a clinic-and-community model for CCSPT. The study will take place over a period of 2.5 years, with a pre-implementation phase of 6 months, an implementation phase of 18 months, and a dissemination phase of 6 months (Fig. 1 and Table 1).

**Table 1:**
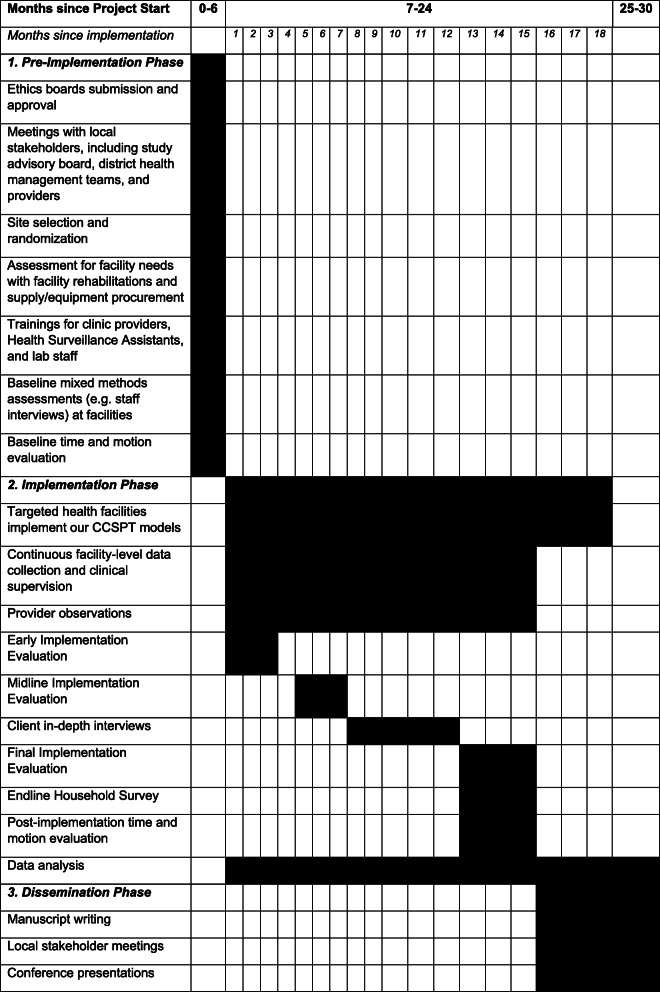
Work plan

The pre-implementation period will be used for obtaining ethical approvals, developing study-specific forms and procedures, selecting and randomizing facilities, and preparing facilities to offer the new services through equipment procurement, facility rehabilitation, and training. We will also complete time-and-motion evaluations and the first round of mixed methods assessments, involving interviews, focus groups, and surveys, to understand the baseline situation. During the implementation period, we will perform additional rounds of mixed methods assessments and collect routine service utilization data from each facility. Assessment details are provided in Table 2. Near the end of the implementation period, we will conduct an Endline Household Survey to assess uptake of CCSPT and FP services among women who live in the catchment areas of the health facilities. The Endline Household Survey will allow us to compare the screening and treatment rates of the clinic-only and the clinic-and-community model.

**Table 2:** Descriptions of assessments, organized by objective

Assessment	Description	Participants	Sample size
Objective 1: Compare the proportion of eligible women who receive CCSPT and FP services in the catchment areas of health facilities assigned to the two models.			
Endline Household Survey	The survey covers prior and recent use of CCSPT and FP services.	Women residing in the catchment areas for the health facilities; must be aged 15–50 years and cannot have had their cervix removed.	8000 women
Objective 2: Assess the acceptability, appropriateness, and feasibility of the two models among key stakeholders, including providers, facility management teams, and clients			
Baseline Assessment Form	The form collects information about the facility’s capabilities for providing CCSPT and FP services.	Completed by research assistants, one form per facility	16 forms (one per facility)
Readiness Assessment Forms	The form includes Likert scale rankings for various parameters of the readiness of the facilities/health system to implement the two models. Participants will answer the survey in small groups, coming to consensus on each ranking.	District health management team members, health facility managers, and community stakeholders	16 forms (one per facility)
Manager and coordinator in-depth interviews (IDIs)	Interviews assess facility managers’ and coordinators’ attitudes towards project implementation.	Facility managers and coordinators currently working at one of the 16 facilities	24 participants (3 from Zomba and 3 from Lilongwe each, at 4 time points)
Provider and HSA focus group discussions (FGDs)	Focus group discussions address providers’ and HSAs’ attitudes towards project implementation, including barriers, facilitators, successes, and challenges.	Providers currently working at one of the 16 facilities; includes HSAs at facilities randomized to community model	16 FGDs, 2 FGDs per district (1 with providers, 1 with HSAs) at 4 time points
Acceptability-Appropriateness-Feasibility tool	This survey is completed by the officer-in-charge and 2 providers at 4 time points and asks participants to rank acceptability, appropriateness, and feasibility of project activities using Likert scales.	1 officer-in-charge and 2 providers from each of the 16 facilities at each time point	Up to 192 forms (up to 48 participants per 4 time points)
Client in-depth interviews (IDIs)	Interviews assess clients’ attitudes towards project implementation, including the acceptability of self-collection and of CCSPT/FP integration.	Clients who participated in cervical cancer screening via self-collection at one of the 16 facilities during the study period	32 participants (16 who were screened at facility and 16 who were screened in community)
Client Exit Survey	This survey includes a 5-point Likert scale to measure clients’ opinions and perceptions on quality of CCSPT and FP services.	Clients who accessed CCSPT and FP services at the facilities	Up to 2370 participants (790 participants each at 3 time points, with the number of participants interviewed per facility proportional to the number of FP clients at that facility)
Workload Assessment Tool	This survey asks providers and lab staff to self-assess their workload.	Providers, HSAs, and lab staff	240 participants (5 per facility per 3 time points)
Observation checklist	Structured observation of providers and lab staff to assess fidelity to project protocols.	Providers and lab staff	Up to 1152 checklists (one per facility per week)
Objective 3: Estimate the cost and budget impact of each model compared to the standard-of-care (VIA and cryotherapy).			
Service utilization data	De-identified information about CCSPT and FP services will be collected from clinic, HSA, and lab registers on a monthly basis.	Providers routinely record visit information in registers; research team members collect data	N/A (difficult to estimate projected number of clients for each register type)
Time and motion study	Research assistants will continuously observe providers, e.g., nurses and HSAs record the time spent on health care activities such as CCSPT and FP including non-health care activities	Providers, nurses, clinicians, HSAs, and lab staff	At least 1 staff member per facility from each category (e.g. provider, HSA, lab staff).
Client Exit Survey	This survey asks clients about the money and time expended accessing CCSPT and FP services at the clinics. It also includes the EQ-5D-3L survey to assess health-related quality-of-life for the cost impact analyses.	Facility clients	Up to 2370 participants (790 participants each at 3 time points, with the number of participants interviewed per facility proportional to the number of FP clients at that facility)

Throughout the implementation period, we will conduct weekly clinical supportive supervision visits and mentor the health facility providers, ensure appropriate logistical support, and assess providers’ fidelity to our protocols. Of note, while our study is ongoing, we will not restrict concomitant care or interventions at the study sites. Specifically, facilities are permitted to continue offering VIA and cryotherapy.

### Setting

This study will occur in two districts in Malawi: (1) Lilongwe in the Central Region and (2) Zomba in the Southern Region. It is unknown how cervical cancer incidence differs across the country, but regional variation in HIV prevalence suggests that there is likely variation in cervical cancer burden, since HIV infection greatly increases risk for persistent HPV infection and cervical cancer development [20]. HIV prevalence among women aged 15–49 is 10.8% nationally, 6.7% in the central region, and 15.7% in the southern region [21]. Without knowledge of how cervical cancer and HPV infection vary geographically, selecting districts from two different regions helps ensure better representativeness.

Malawi is a country of 18.1 million people in southeastern Africa. Gross domestic product (GDP) is $516 USD, among the lowest in the world [22]. Health care facilities in Malawi are operated by the Ministry of Health (MoH), the private for-profit sector, and the Christian Health Association of Malawi (CHAM), which is Christian-affiliated and heavily publicly subsidized [23]. In most facilities regardless of sector, service utilization data is recorded in standardized MoH registers. The MoH organizes regular surveillance of facility service utilization by having facilities submit monthly reporting forms to District Health Offices [23].

HSAs in Malawi comprise 30% of the health care workforce [23]. Originally, HSAs focused on immunizations and health education, but their role has since been expanded to clinical activities such as pediatric disease management, HIV testing, and FP provision [24]. HSAs typically deliver services in community outreach or village clinics, which are held in a given location anywhere from several times a week to once every few weeks. Some HSAs are utilized to provide basic care in health facilities or to provide home visits. The minimum qualifications for an HSA are a secondary school certificate and completion of a 12-week MoH-administered HSA training program [24].

In 2015, there were 130 public health facilities providing VIA and one providing Pap smear [3]. For treatment, 32 public facilities provided cryotherapy, though the machines were only functional at 22, and 11 offered thermal ablation. More sites have likely added thermal ablation since 2015, since thermal ablation was only introduced in the country in 2013 and added to the National Cervical Cancer Control Strategy in 2016 [13].

### Sample size calculations

Sample size calculations were based on the ability to detect a difference in service uptake through the Endline Household Survey. As noted earlier, only 26.5% of Malawian women had been screened at least once in their lifetime as of 2015 with use of VIA for primary screening [3], which is much lower than the targeted rate of 80% set at the onset of Malawi’s Cervical Cancer Control Program in 2004 [16]. We estimated that with the introduction of HPV self-sampling in our clinic-only arm, at least 40–50% of eligible women in our facility catchment areas will be screened during our 18 months of implementation. We then estimated that with the addition of HPV self-sampling in the community to reach rural women, we would be able to approach Malawi’s targeted rate of 80% of eligible women screened in our clinic + community arm. Based on data obtained from potential facilities in Lilongwe and Zomba, we also estimated an average cluster size of 8000 eligible women per health facility and a coefficient of variation of the cluster sizes of 0.35. Finally, we estimated that our intra-cluster correlation coefficient (ICC) will be between 0.12 and 0.19, based on other similar studies performed in Malawi, where the ICC ranged from 0.004 to 0.20 [25, 26]. With 16 randomized facilities (8 per arm), we initially estimated that we would have at least 80% power to detect a difference of at least 25–30% between our 2 arms, even if the proportion of women screened in the clinic-only arm is as low as 40%. For example, if the clinic-only arm found that 40% of women had been screened, we would have 80% power to detect a difference if the clinic + community arm had screened at least 75% of women in their catchment areas.

However, after performing site selection and randomization, we learned that the two central/district hospitals selected could not be assigned to model 2 as their HSAs did not perform community-based services. Therefore, we dropped these 2 hospitals from our sample size calculations and decreased our average cluster size to 3400 eligible women accordingly. With an ICC of 0.15 and coefficient of variation of cluster sizes of 0.35, we will have at least 83% power to detect of difference of at least 30% between our 2 arms (with 7 facilities in each arm), even if the proportion of women screened in the clinic-only arm is as low as 40%. All power calculations were calculated in R (Version 3.5.1, clusterPower package).

For the other study components, the sample size explanations are listed in Table 2.

### Site selection and randomization

In each district, we selected 8 facilities that were representative of the 4 facility types in which our models would most likely be implemented: (1) one central/district hospital, (2) one CHAM hospital, (3) two urban health centers, and (4) four rural health centers. The facilities were chosen in consultation with the District Health Management teams of each district. We evaluated the readiness of each facility to implement our models, excluding those with no existing FP services or laboratories. We discussed potential facilities with other local partners, including other research groups, nonprofits, and international health organizations.

Once the 16 facilities were selected, they were assigned a code. The facilities were then randomized to one of the models in a 1:1 randomization within each of the 4 facility type strata (Table 3) by a UNC Project-Malawi biostatistician, using the facility codes. This randomization resulted in 8 health facilities assigned to model 1 (4 in Lilongwe, 4 in Zomba) and 8 health facilities assigned to model 2 (4 in Lilongwe, 4 in Zomba). However, when we discovered that neither Bwaila District Hospital in Lilongwe nor Zomba Central Hospital in Zomba could be randomized to model 2 given their lack of HSAs (see sample size text above), we determined that we could not include Zomba Central Hospital or Bwaila District Hospital in our analyses to compare model 1 and model 2 (see “Sample size calculations” section). Therefore, we will only be analyzing 14 health facilities for that comparison: 7 assigned to model 1 (3 in Lilongwe, 4 in Zomba) and 7 assigned to model 2 (4 in Lilongwe, 3 in Zomba).

**Table 3:** Selected sites with their initially assigned model number, within their health facility stratum

Health facility strata	Lilongwe district	Assigned model	Zomba district	Assigned model
**Central/district hospital**	Bwaila District Hospital	1	Zomba Central Hospital	2^a^
**CHAM hospital**	Nkhoma Hospital	2	St. Luke’s Hospital	1
**Urban health center**	Kawale Health Center	2	Matawale Health Center	2
	Area 18 Health Center	1	Zomba City Clinic	1
**Rural health facility**	Kabudula Rural Hospital	1	Domasi Rural Hospital	2
	Lumbadzi Health Center	2	Namasalima Health Center	1
	Chileka Health Center	2	Ngwelero Health Center	1
	Chiwamba Health Center	1	Likangala Health Center	2

*CHAM* Christian Association of Malawi

^a^Bwaila District and Zomba Central Hospitals will not be included in the final comparison of randomized arms as these hospitals did not have health surveillance assistants who could provide community-based services for model 2

### Facility preparation

To facilitate facilities’ ability to deliver CCSPT services per our algorithm, we will conduct needs assessments at each selected site and organize procurements and facility rehabilitation. We will modify existing MoH registers for facility CCSPT, facility FP, and community FP services to capture information on the new services. In partnership with MoH, we will design new registers for HPV lab testing and community cervical cancer screening. We will organize technical training and project-specific training sessions for clinic providers, HSAs, and laboratory staff. These will cover how to give instructions for self-sampling (for both providers and HSAs), how to perform thermal ablation (for providers only), and how to conduct GeneXpert HPV testing (laboratory staff), as well as project-specific procedures for documentation.

### Description of models

In both model 1 and model 2, through group educational talks and one-on-one counseling, women will be educated about cervical cancer screening and the opportunity to perform self-sampling for HPV testing. The facility staff will be encouraged to hold the CCSPT educational talks in waiting rooms for the FP clinic, antiretroviral therapy (ART) clinic, and other clinics.

Once a woman presents to the CCSPT clinic at the facility, she will be offered self-collection if she meets the following eligibility criteria: (1) aged 25–49 years old, (2) no history of total hysterectomy, (3) no history of cervical cancer, and (4) not currently pregnant. Self-collected samples will then be sent to the facility laboratory for high-risk HPV (hrHPV) testing using the GeneXpert® (Cepheid Inc, Sunnyvale, CA, USA) platform. We chose to use the Xpert HPV assay because it offers several advantages over other commercially available HPV assays: results are available within 1–2 h, all reagents are contained in one cartridge, and laboratory personnel only need a short training course to use the machines. In addition, the Xpert HPV assay has been validated in Malawi, and many facilities in Malawi have already invested in Xpert machines for tuberculosis diagnostic testing [27].

Women who screen positive for hrHPV at the clinic will be offered same-day VIA to determine whether they are eligible for ablative treatment. Eligibility for thermal ablation requires that (1) no cervical lesions or any visible lesion covers <75% of the cervix and can be seen in its entirety, (2) the lesion does not extend into the endocervical canal or to the vaginal wall, (3) the squamocolumnar junction is visible, (4) the client is more than 3 months postpartum if recently pregnant, (5) there is no suspicion of cervical cancer, (6) there are no polyps or scarring impeding contact of the probe, and (7) there is no evidence of current menstruation or cervicitis. HPV-positive and treatment-eligible women will be offered thermal ablation on the same day of screening.

**Fig. 2:**
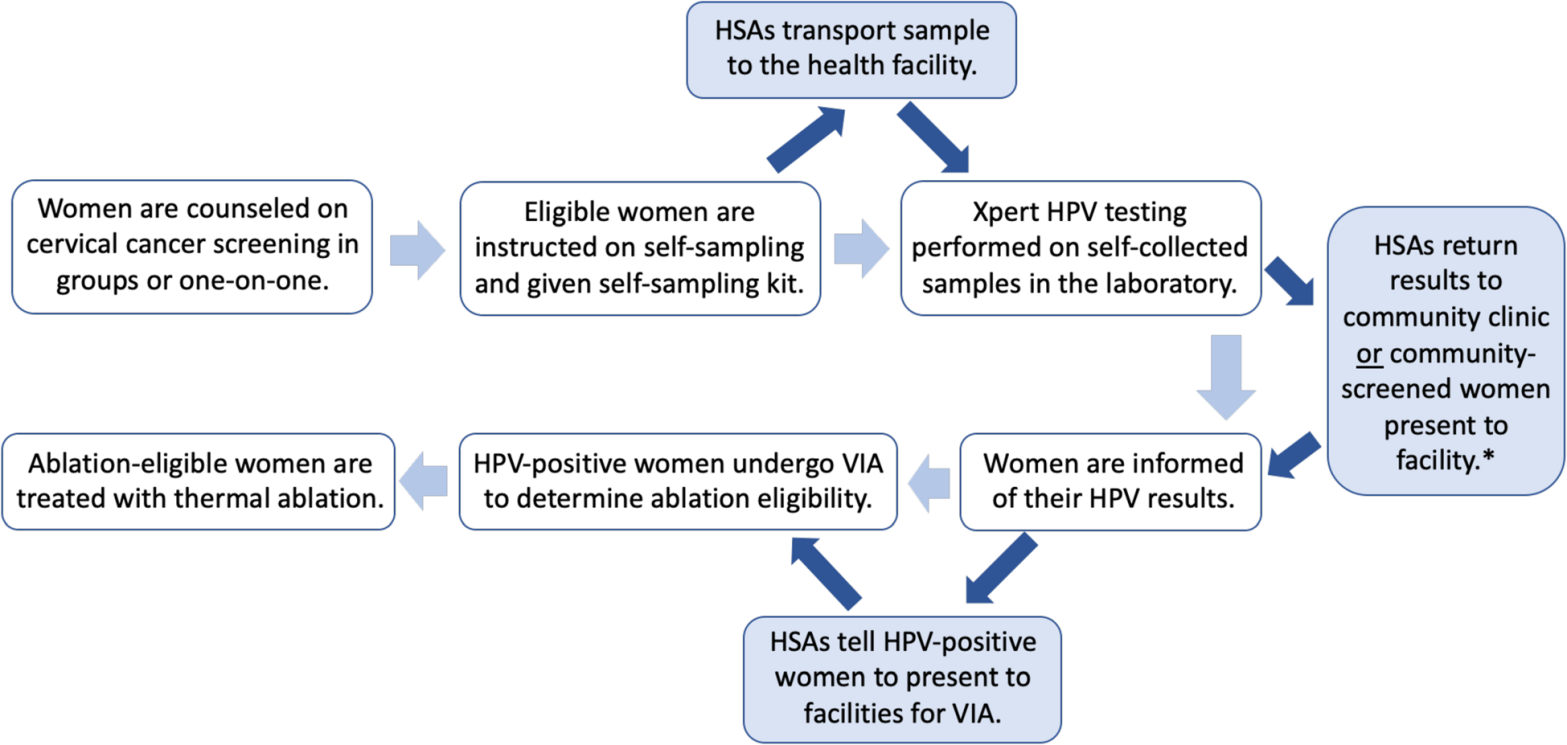
Patient flow for screening and treatment. Blue boxes and darker arrows indicate the community components that will only be present in model 2. *Note that result return will be delayed for community-screened women; same-day results will only be possible for women screened at facilities. HSA, health surveillance assistant

In model 2, women living in the health facility’s catchment area will also be offered self-sampling in the community. HSAs and other health facility staff will give educational talks about self-sampling to groups of women at village and outreach clinics. Interested women can perform self-sampling on the day of the community clinic, and HSAs will transport the sample back to the health facility for testing. Women can receive results at the next community clinic or by presenting to the associated health facility. If any women screens HPV-positive, she will be referred for VIA and possible treatment. Patient flow is illustrated in Fig. 2, with light blue shading for the community-based components.

Educational talks and self-sampling instructions can be given by HSAs, nurses, mid-level providers, and doctors, while only nurses, mid-level providers, and doctors can perform VIA and thermal ablation. All clinical staff will undergo protocol-specific training sessions before conducting duties related to the new interventions. They will also receive weekly visits from the study’s clinical mentors (either clinicians or nurses), who will observe the clinical staff using observation checklists and collect data from the clinic and HSA registers on a monthly basis to promote adherence to study protocols and accurate data entry into the registers.

### Assessments

#### Overview of study components

Details of assessments, including how they align with objectives, are shown in Table 2. For the assessments with specific eligibility criteria for participants, the criteria are listed in the “Participants” column in the table. Most quantitative data collection will be conducted using the Open Data Kit (ODK) software on electronic tablets.

#### Endline Household Survey

The Endline Household Survey, conducted at months 13–15 after implementation, will be the primary tool to assess the uptake of CCSPT and FP services among women in the catchment areas of the health facilities. Households will be sampled in a two-stage sampling design. Stage 1 will include a random sample of enumeration areas (EAs) from all of the facilities’ catchment areas. This random sample will be selected with the number of EAs proportional to the size of the catchment area. In each selected EA, we will undertake a household listing to create a sampling frame of households for the survey. In stage 2, a random sample of households will be selected. Our target sample size is up to 8000 women aged 15–50 years from the 16 health facility catchment areas. The age range is intended to include women most likely to utilize FP services, as well as those in the target range for CCSPT. With the expectation of ~1.1 women aged 15–50 in our households, the sample will include ~8000 households selected from 266 EAs selected proportionally from facility catchment areas (randomly selecting ~30 households/EA). Parental consent will be obtained for participants 15–17 years old, as described in the “Ethical considerations” section. If a randomly sampled woman does not consent for the survey, she will be replaced with another randomly selected woman from her EA.

Women aged 15–50 years will be eligible to participate in the interviewer-administered survey, provided that they have not had their cervix removed. The survey will cover demographic information, reproductive health information, HIV status, distance to the nearest health facility, prior FP use, FP use during project implementation, prior CCSPT services received, and CCSPT services received during project implementation.

#### Mixed methods assessments

To assess acceptability, appropriateness, and feasibility, we will conduct mixed methods assessments at multiple time points. Pre-implementation, we will perform baseline assessments at each facility and administer readiness assessment surveys to small groups of key stakeholders, including representatives from MoH district health management teams and community leaders. We will also conduct the first round of in-depth interviews (IDIs) with facility managers and coordinators and focus group discussions (FGDs) with clinic providers and HSAs. Each IDI/FGD participant will complete a survey before the discussion in which they rank the acceptability, appropriateness, and feasibility of the interventions.

During implementation, we will conduct three rounds of implementation evaluations at early (0–3 months after implementation), midline (5–7 months), and final (13–15 months) time points. These mixed methods assessments will include manager IDIs, provider FGDs, acceptability-appropriateness-feasibility surveys, Client Exit Surveys, and Workload Assessment Surveys. We will conduct client IDIs in months 7–13 after implementation. Throughout implementation, the study team will use an observation checklist to determine how closely facility staff is adhering to our interventions. All surveys will be administered by interviewers.

At the end of the pre-implementation assessment and each round of mixed methods assessments, the study team will convene stakeholders meetings at district and facility levels to validate the findings and seek explanation from the stakeholders on the potential reasons for our successes or failures. Any recommendations from the stakeholders about how to modify study interventions will be documented.

#### Cost and budget impact

Cost and budget information will be obtained from multiple sources, including the time-and-motion evaluations (conducted pre- and post-implementation), the Client Exit Survey, the Workload Assessment Tool, project reports, and account expenditure reports. Key activities that will be costed include provider wages and time, health worker training and briefings, community sensitization, development of project materials, and patient costs.

### Analysis plan

#### Endline Household Survey

Key outcomes from the Endline Household Survey include (1) the proportion of eligible women who have received CCS during the 18 months of study implementation, (2) the proportion of treatment-eligible women who received thermal ablation during the study period, and (3) the proportion of women aged 15–50 years who are using a modern FP method. These proportions will be calculated using descriptive statistics. Differences between the clinic-only and clinic-and-community models will be assessed via logistic regression, adjusted for district, facility size, and facility type. The regression model will account for health facility clustering, and analyses will be adjusted for the small number of clusters [28, 29].

#### Mixed methods assessments

We will analyze the findings from mixed methods assessments using the normalization process theory to gauge potential sustainability of the interventions [30].

The Baseline Assessment Tool and Readiness Assessment Form, administered pre-implementation, will be analyzed to identify any significant differences at baseline. At endline analysis, we will assess the association between pre-implementation preparedness and the primary outcomes of the intervention. The Baseline Assessment Tool will yield a mix of continuous variables (e.g., number of health workers) and dichotomous variables (e.g., availability of equipment). Meanwhile, the Readiness Assessment Tool will provide an average Likert score for variety of parameters related to readiness, as ranked by the groups of stakeholders who complete the form for each facility.

The IDIs and FGDs are primarily designed to assess the key stakeholder views’ on the project’s implementation, including perceived successes and challenges and reasons for each. Content analysis of the IDIs and FGDs will be done using qualitative analysis software. From the acceptability-appropriateness-feasibility survey, average Likert scale scores for acceptability, appropriateness, and feasibility will be calculated for each facility and each model. Any large variations across the types of respondents will be noted. Qualitative data will be triangulated with quantitative data to provide explanations for facilities scoring below or above average on these assessments. A trend graph of acceptability and feasibility aggregate scores at each phase of the project will be plotted for each health facility at the completion of the study.

Client satisfaction will be measured on the Client Exit Survey using Likert scales and open-ended questions. Clients who score at least an average of 4 on the Likert scale will be classified as satisfied, and proportion of satisfied FP and CCSPT clients will be calculated for each facility. Responses to the open-ended questions will be analyzed using inductive thematic content analysis to provide explanations for clients’ satisfaction or non-satisfaction.

We will assess any changes from baseline in the service providers’ workload by estimating the daily client-to-provider ratios and reviewing self-reported assessment of the providers’ workload on the workload assessment tool. Findings from the Workload Assessment Tool will provide context for variations in the uptake of CCSPT and FP and will help us understand how non-CCSPT services have been affected at the facility by the introduction of our CCSPT intervention.

On a regular basis, the project staff will observe facility staff as they deliver CCSPT and FP services and will record adherence to protocols using the Observation Checklist. Staff will be aware of the presence of observers and instructed to continue performing duties as they ordinarily would; the observations are intended to be overt and non-participatory. Facilities will be graded in the poor, average, or well-performing categories based on the average scores from observations of their staff. We will compare the scores across time points, facilities, and models.

#### Cost and budget impact

For the CCSPT intervention proposed, we will calculate the incremental cost of the two models (including both costs incurred and averted), the cost per woman screened, and the cost per cervical cancer case averted. We will also estimate the cost per use of CCSPT and FP services. Cost data will be used to conduct a budget impact analysis from the Malawi MoH perspective.

Micro-costing studies will use the time and motion evaluations, routine service utilization data, study budgets, workload assessments, structured observations, and client exit surveys. Estimated costs will also be abstracted from published government reports and the health economics literature, based on methods from published cost-effectiveness research [31, 32]. Costs will be compared between the two models and between each model and the current standard of care (VIA and cryotherapy). The number of women screened will be based on the results of the Endline Household Survey.

To estimate the impact of HPV screening and treatment, we will use a decision analysis model to simulate HPV infection, clearance, persistence, and progression to high-grade cervical intraepithelial lesion and cancer (CIN2+), informed by previous modeling work [33, 34]. Our modeling will also capture differences in cost and outcomes by HIV status.

For budget impact analysis, we will consider direct program costs, to ensure that measurements of MoH costs reflect the opportunity cost of the resources used in delivering services. The primary analysis will be from the programmatic perspective, the point of view of decision makers. A secondary analysis will be presented from the societal perspective, using data on economic productivity for disease averted and costs of accessing services.

### Ethical considerations

This study has been approved by the University of North Carolina Institutional Review Board (IRB#: 19-0638) and the National Health Sciences Research Committee of Malawi (NHSRC#: 19/03/2255). All protocol modifications will be communicated to these regulatory boards, and protocol modifications relevant to health care provision will be communicated to study sites. The currently approved version of the protocol is Version 2.0, dated September 6, 2019, and is the version presented here.

Written informed consent will be obtained for participants in the Endline Household Survey, IDIs, and FGDs. This will include women living in the catchment areas of the facilities who are randomly sampled for the Endline Household Survey, facility managers, providers, and HSAs, and a small selection of CCSPT clients. Adolescents may be sampled for Endline Household Survey because women aged 15–50 years are eligible. For any potential participants aged 15–17 years, we will require both pediatric assent and parental consent for enrollment. IDI/FGD sessions will be conducted by experienced qualitative researchers, who are accustomed to leading sensitive discussions.

Research participants will be identified only by a unique study identification code on all records. We expect that any adverse events related to study participation will be social harms, and we do not anticipate any serious adverse events. Any study site staff member or research team member can report protocol deviations or social harms to the study coordinator who will record them in the *Protocol Deviations and Social Harms Log*, which will be submitted to the IRBs on an annual basis. Because risks to participants are minimal, a data and safety monitoring board will not be required for this study.

The requirement for informed consent was waived by the ethical committees for all assessments besides those listed above as they do not involve collection of identifiable data. All data is securely stored on the UNC OneDrive on the UNC server, and only research staff who have received ethical committee approval have access to it through their private email address and password. Data collected from the registers is double entered into the database to minimize data entry errors. The final trial datasets will be uploaded to USAID’s Development Data Library for open access.

## Discussion

Malawi has struggled to achieve widespread CCSPT coverage and carries a disproportionate burden of cervical cancer. Given recent advances in technology, including the Xpert HPV assay, HPV-based screening programs are more feasible to implement in resource-limited settings, and the stability of self-collected cervicovaginal samples opens up the opportunity for community-based screening. Meanwhile, thermal ablation provides a less resource-intensive alternative to cryotherapy for treatment. Our cluster randomized trial will provide key insights into the effectiveness, acceptability, appropriateness, feasibility, and budget impact of clinic-only and community-and-clinic models for implementing HPV-based screening in Malawi. We expect the clinic-and-community model to be more effective, acceptable, and appropriate. Community-based services may be more difficult and expensive to implement than clinic-only services, but we hypothesize that community screening will still be feasible in Malawi and more cost-effective than the current standard-of-care. Community programs also uniquely hold the potential to reduce disparities in screening and improve health equity.

Studies in other low-resource countries have demonstrated that training community health workers like HSAs to offer self-sampling is feasible and has the potential to improve screening coverage. In addition, the WHO now recommends self-testing options for sexual and reproductive health, including HPV self-sampling for primary cervical cancer screening [4]. A cluster randomized study in Argentina demonstrated a four-fold increase in screening rates with community health workers offering HPV self-sampling, compared with community health workers counseling women to undergo clinician-sampling at a facility [18]. In Uganda, screening was higher with community-based HPV self-sampling compared to the control of clinic-based VIA screening, in preliminary results from a cluster randomized trial [19]. The same project showed that community-based HPV self-sampling was more cost-effective than clinic-based VIA screening, with a wide margin for increasing costs of the HPV-based program [35]. Our study will investigate the feasibility of community self-sampling in Malawi and provide a direct comparison to facility-only services. Using stratified randomization further allows us to assess implementation success in a variety of settings, including urban and rural, district-level and local, and public and private facilities.

Linking community-screened women with appropriate care is crucial for cervical cancer prevention and can be especially challenging with community-based screening. In the pilot results of a Uganda trial comparing community HPV screening versus clinic-based VIA screening, over 50% of 73 women who were positive for hrHPV after community self-sampling could not be reached by telephone for result delivery [19]. Loss to follow-up is already a concern with the current standard of care in Malawi: only 43% of women who are VIA-positive receive appropriate treatment [3]. Our study will provide insight into whether appropriate follow-up is feasible with community-based screening.

Studies never perfectly approximate real-world settings, but we have tried to increase fidelity by working closely with facilities and MoH partners to ensure that we are implementing only interventions that could be realistically maintained by these partners after the study period. This approach could potentially become challenging as there could be expectation from providers for incentives, which if implemented would make our study vulnerable to unrealistic conclusions about the feasibility of our interventions.

Disseminating findings to local partners is a priority for our team, since we hope that our study results will directly inform policy in Malawi. Key stakeholders, including the MoH, will already be engaged with our project by serving on our project’s advisory board and attending our regular stakeholder meetings which are intended primarily to validate our findings and seek explanations for successes and failures. After project completion, we plan to hold a large dissemination meeting with relevant policy makers, academic institution representatives, and private health leaders. To contribute to the larger body of evidence on context-appropriate CCSPT solutions, we will present our findings through peer-reviewed journals and regional and international conferences as per the CONSORT 2010 statement for randomized pilot and feasibility trials [36]. We will also present our findings to the MoH committees involved in family planning and CCSPT services. Progression of implementation of our interventions will depend on the input and agreement of our key stakeholders, including these MoH committees, as well as the availability of funding to pay for HPV cartridges once funding for our trial ends.

In accordance with USAID’s Operational Policy Automated Directives System Series 500, Chapter 579, and the newly approved Public Access Plan, the research team will submit the study dataset using the platform-independent and non-proprietary comma-separated values (also known as [CSV]) format. The study dataset will be submitted to USAID’s Data Development Library within 30 calendar days after the dataset is first used to produce an intellectual work. For the purpose of this project, “Intellectual Work” is defined as the initial online or print publication of the primary study manuscript. If 30 days is not feasible, the research team will consult with the USAID Agreement Officer Representative to determine a suitable time frame for dataset submission.

## Data Availability

Not applicable

## Abbreviations

CCSPT: Cervical cancer screening and preventive treatment
CHAM: Christian Health Association of Malawi
FGD: Focus group discussion
FP: Family planning
GDP: Gross domestic product
HPV: Human papillomavirus
HSA: Health surveillance assistant
ICC: Intra-cluster correlation coefficient
IDI: In-depth interview
MoH: Ministry of Health
ODK: Open Data Kit
USD: United States dollar
VIA: Visual inspection with acetic acid
WHO: World Health Organization

## References

[1] International Agency for Cancer Research (FR). Malawi fact sheet, 2019 [Internet]. World Health Organization: 2019 May. https://gco.iarc.fr/today/data/factsheets/populations/454-malawi-fact-sheets.pdf. Accessed 13 May 2020.

[2] Arbyn M, Weiderpass E, Bruni L, de Sanjose S, Saraiya M, Ferlay J (2020). Estimates of incidence and mortality of cervical cancer in 2018: a worldwide analysis. Lancet Glob Health..

[3] Msyamboza KP, Phiri T, Sichali W, Kwenda W, Kachale F (2016). Cervical cancer screening uptake and challenges in Malawi from 2011 to 2015: retrospective cohort study. BMC Public Health..

[4] World Health Organization (CH). WHO guidelines for screening and treatment of precancerous lesions for cervical cancer prevention. South Africa: WHO Africa; 2013. 40p. https://www.who.int/reproductivehealth/publications/cancers/screening_and_treatment_of_precancerous_lesions/en. Accessed 13 May 2020 24716265

[5] Mustafa RA, Santesso N, Khatib R, Mustafa AA, Wiercioch W, Kehar R, Gandhi S, Chen Y, Cheung A, Hopkins J, Ma B, Lloyd N, Wu D, Broutet N, Schünemann HJ (2016). Systematic reviews and meta-analyses of the accuracy of HPV tests, visual inspection with acetic acid, cytology, and colposcopy. Int J Gynaecol Obstet..

[6] Cuzick J, Cuschieri K, Denton K, Hopkins M, Thorat MA, Wright C, Cubie H, Moore C, Kleeman M, Austin J, Ashdown-Barr L, Hunt K, Cadman L (2015). Performance of the Xpert HPV assay in women attending for cervical screening. Papillomavirus Res..

[7] Rosenbaum AJ, Gage JC, Alfaro KM, Ditzian LR, Maza M, Scarinci IC, Felix JC, Castle PE, Villalta S, Miranda E, Cremer ML (2014). Acceptability of self-collected versus provider-collected sampling for HPV DNA testing among women in rural El Salvador. Int J Gynaecol Obstet..

[8] Yeh PT, Kennedy CE, de Vuyst H, Narasimhan M (2019). Self-sampling for human papillomavirus (HPV) testing: a systematic review and meta-analysis. BMJ Glob Health..

[9] Lin C, Zeng X, Cui J, Liao G, Wu Z, Gao Q (2017). Stability study of cervical specimens collected by swab and stored dry followed by human papillomavirus DNA detection using the cobas 4800 test. J Clin Microbiol..

[10] Ejegod DM, Pedersen H, Alzua GP, Pedersen C, Bonde J (2018). Time and temperature dependent analytical stability of dry-collected Evalyn HPV self-sampling brush for cervical cancer screening. Papillomavirus Res..

[11] Randall TC, Sauvaget C, Muwonge R, Trimble EL, Jeronimo J (2019). Worthy of further consideration: an updated meta-analysis to address the feasibility, acceptability, safety and efficacy of thermal ablation in the treatment of cervical cancer precursor lesions. Prev Med..

[12] Pinder LF, Parham GP, Basu P, Muwonge R, Lucas E, Nyambe N, Sauvaget C, Mwanahamuntu MH, Sankaranarayanan R, Prendiville W (2020). Thermal ablation versus cryotherapy or loop excision to treat women positive for cervical precancer on visual inspection with acetic acid test: pilot phase of a randomised controlled trial. Lancet Oncol..

[13] Malawi Ministry of Health (2016). National Cervical Cancer Prevention Program Strategy.

[14] Parkin DM, Whelan SL, Ferlay J, Teppo L, Thomas DB (2004). Cancer incidence in five continents, volume VIII.

[15] High-Impact Practices in Family Planning (HIPs) (2015). Community health workers: bringing family planning services to where people live and work.

[16] National Statistical Office (NSO) and ICF (2017). Malawi demographic and health survey 2015-16.

[17] Lemani C, Tang JH, Kopp D, Phiri B, Kumvula C, Chikosi L, Mwale M, Rosenberg NE (2017). Contraceptive uptake after training community health workers in couples counseling: a cluster randomized trial. PLoS One..

[18] Arrossi S, Thouyaret L, Herrero R, Campanera A, Magdaleno A, Cuberli M, Barletta P, Laudi R, Orellana L, EMA Study team (2015). Effect of self-collection of HPV DNA offered by community health workers at home visits on uptake of screening for cervical cancer (The EMA Study): a population-based cluster-randomised trial. Lancet Glob Health..

[19] Moses E, Pedersen HN, Mitchell SM, Sekikubo M, Mwesigwa D, Singer J, Biryabarema C, Byamugisha JK, Money DM, Ogilvie GS (2015). Uptake of community-based, self-collected HPV testing vs. visual inspection with acetic acid for cervical cancer screening in Kampala, Uganda: preliminary results of a randomised controlled trial. Trop Med Int Health..

[20] Rohner E, Butikofer L, Schmidlin L, Sengavi M, Maskew M, Giddy J (2020). Cervical cancer risk in women living with HIV across four continents: a multicohort study. Int J Cancer..

[21] The World Bank. Malawi [Internet]. 2020. https://data.worldbank.org/country/malawi. Accessed 13 May 2020.

[22] Africa Health Workforce Observatory. Human resources for health country profile, Malawi. 2009 Oct. 55p. https://ecsahc.org/wp-content/uploads/2017/05/malawi_country_profile-Human-Resources.pdf. Accessed 13 May 2020.

[23] Smith S, Deveridge A, Berman J, Negan J, Mwambene N, Chingaipe E (2014). Task-shifting and prioritization: a situational analysis examining the role and experiences of community health workers in Malawi. Hum Resour Health..

[24] Mwapasa V, Joseph J, Tchereni T, Jousset A, Gunda A (2017). Impact of mother-infant pair clinics and short-text messaging service (SMS) reminders on retention of HIV-infected women and HIV-exposed infants in eMTCT care in Malawi: a cluster randomized trial. J Acquir Immune Defic Syndr..

[25] Dovel K, Shaba F, Nyirenda M, Offorjebe OA, Balakasi K, Phiri K, Nichols B, Tseng CH, Bardon A, Ngona K, Hoffman R (2018). Evaluating the integration of HIV self-testing into low-resource health systems: study protocol for a cluster-randomized control trial from EQUIP innovations. Trials..

[26] Cuschieri K, Geraets D, Cuzick J, Cadman L, Moore C, Vanden Broeck D (2016). Performance of a cartridge-based assay for detection of clinically significant human papillomavirus (HPV) infection: lessons from VALGENT (Validation of HPV Genotyping Tests). J Clin Microbiol..

[27] Cubie HA, Morton D, Kawonga E, Mautanga M, Mwenitete I, Teakle N, Ngwira B, Walker H, Walker G, Kafwafwa S, Kabota B, ter Haar R, Campbell C (2017). HPV prevalence in women attending cervical screening in rural Malawi using the cartridge-based Xpert® HPV assay. J Clin Virol..

[28] Zeger SL, Liang K, Albert PS (1988). Models for longitudinal data: a generalized estimating equation approach. Biometrics..

[29] Li P, Redden DT (2015). Small sample performance of bias-corrected sandwich estimators for cluster-randomized trials with binary outcomes. Stat Med.

[30] May CR, Cummings A, Girling M, Bracher M, Mair FS, May CM, Murray E, Myall M, Rapley T, Finch T (2018). Using Normalization Process Theory in feasibility studies and process evaluations of complex healthcare interventions: a systematic review. Implement Sci..

[31] Smith JA, Sharma M, Levin C, Baeten JM, van Rooyen H, Celum C, Hallett TB, Barnabas RV (2015). Cost-effectiveness of community-based strategies to strengthen the continuum of HIV care in rural South Africa: a health economic modelling analysis. Lancet HIV..

[32] Golovaty I, Sharma M, Van Heerden A, van Rooyen H, Baeten JM, Celum C (2018). Cost of integrating non-communicable disease screening into home-based HIV testing and counseling in South Africa. J Acquir Immune Defic Syndr..

[33] Barnabas RV, Laukkanen P, Koskela P, Kontula O, Lehtinen M, Garnett GP (2006). Epidemiology of HPV 16 and cervical cancer in Finland and the potential impact of vaccination: mathematical modelling analyses. PLoS Med..

[34] Tan N, Sharma M, Winer R, Galloway D, Rees H, Barnabas RV (2018). Model-estimated effectiveness of single dose 9-valent HPV vaccination for HIV-positive and HIV-negative females in South Africa. Vaccine.

[35] Mezei AK, Pedersen HG, Sy S, Regan C, Mitchell-Foster SM, Byamugisha J (2018). Community-based HPV self-collection versus visual inspection with acetic acid in Uganda: a cost-effectiveness analysis of the ASPIRE trial. BMJ Open..

[36] Eldridge SM, Chan CL, Campbell MJ, Bond CM, Hopewell S, Thabane L (2016). CONSORT 2010 statement: extension to randomised pilot and feasibility trials. Pilot Feasibility Stud.

